# Viral Evasion of the Complement System and Its Importance for Vaccines and Therapeutics

**DOI:** 10.3389/fimmu.2020.01450

**Published:** 2020-07-09

**Authors:** Jack Mellors, Tom Tipton, Stephanie Longet, Miles Carroll

**Affiliations:** ^1^Public Health England, National Infection Service, Salisbury, United Kingdom; ^2^Department of Infection Biology, Institute of Infection and Global Health, University of Liverpool, Liverpool, United Kingdom

**Keywords:** virology, immunology, complement system, innate immunity, vaccines, therapeutics

## Abstract

The complement system is a key component of innate immunity which readily responds to invading microorganisms. Activation of the complement system typically occurs via three main pathways and can induce various antimicrobial effects, including: neutralization of pathogens, regulation of inflammatory responses, promotion of chemotaxis, and enhancement of the adaptive immune response. These can be vital host responses to protect against acute, chronic, and recurrent viral infections. Consequently, many viruses (including dengue virus, West Nile virus and Nipah virus) have evolved mechanisms for evasion or dysregulation of the complement system to enhance viral infectivity and even exacerbate disease symptoms. The complement system has multifaceted roles in both innate and adaptive immunity, with both intracellular and extracellular functions, that can be relevant to all stages of viral infection. A better understanding of this virus-host interplay and its contribution to pathogenesis has previously led to: the identification of genetic factors which influence viral infection and disease outcome, the development of novel antivirals, and the production of safer, more effective vaccines. This review will discuss the antiviral effects of the complement system against numerous viruses, the mechanisms employed by these viruses to then evade or manipulate this system, and how these interactions have informed vaccine/therapeutic development. Where relevant, conflicting findings and current research gaps are highlighted to aid future developments in virology and immunology, with potential applications to the current COVID-19 pandemic.

## Introduction

The complement system is a heat-labile component of native plasma involving both extracellular and cell surface membrane-associated proteins which form a major constituent of the innate immune response. The whole system is comprised of over 30 proteins which have the potential to react via an enzymatic cascade in response to recognition of various stimuli, such as pathogen-associated molecular patterns (PAMPs) and abnormal or damaged host cells. Activation of the complement system typically occurs via three distinct target recognition pathways (the classical, lectin, and alternative pathways) which converge at a single point; the cleavage of complement component 3 (C3). Each pathway has its own unique protease zymogens to recognize and respond to different antigens, but all pathways primarily work to: opsonise pathogens, lyse pathogens and infected cells, regulate the inflammatory response, and enhance the clearance of immune complexes and cell debris (1, 2).

In the context of viral infections, the complement system has been shown to exhibit numerous antiviral mechanisms via direct neutralization of both enveloped and non-enveloped viruses, and/or the promotion of other immune responses. The complement system can directly neutralize virus particles through opsonisation (3), membrane attack complex (MAC) formation on the virion (4), MAC formation on virus-infected cells (5), or targeting of intracellular viral components for proteasomal degradation (6). Other immune responses may also be modulated by the complement system to promote viral clearance, including: the regulation of inflammation/chemotaxis (7), the induction of the antiviral state (6), and the enhancement of adaptive immune responses specific to viral antigens (8, 9). The effectiveness and outcome of this response can vary depending on the infectious agent and host genetic variability.

### Classical Complement Pathway

The classical complement pathway is typically activated when hexameric C1q proteins bind to the fragment crystallisable (FC) CH2-domains of antigen-bound IgM and/or IgG immune complexes (10–12). The binding affinity of C1q to IgG is dependent on the IgG isotype with the greatest affinity for IgG-3, then IgG-1, a weak association with IgG-2, and no interaction with IgG-4 (13). However, for downstream activation and complement lysis activity, the response is more efficient following IgG1-C1q interactions rather than IgG3-C1q interactions (14). C1q is also a versatile pattern recognition molecule. In absence of antibodies, C1q can directly bind to apoptotic cells (15) or proteins on the cell-surface membrane of some pathogens, such as human immunodeficiency virus (HIV) (16) and dengue virus (DENV) (17). C1q can also bind other host plasma proteins such as C-reactive protein (18), fibronectin (19, 20), decorin (21), lactoferrin (22), pentraxin-3 (23), and serum amyloid P component (24).

**Figure 1:**
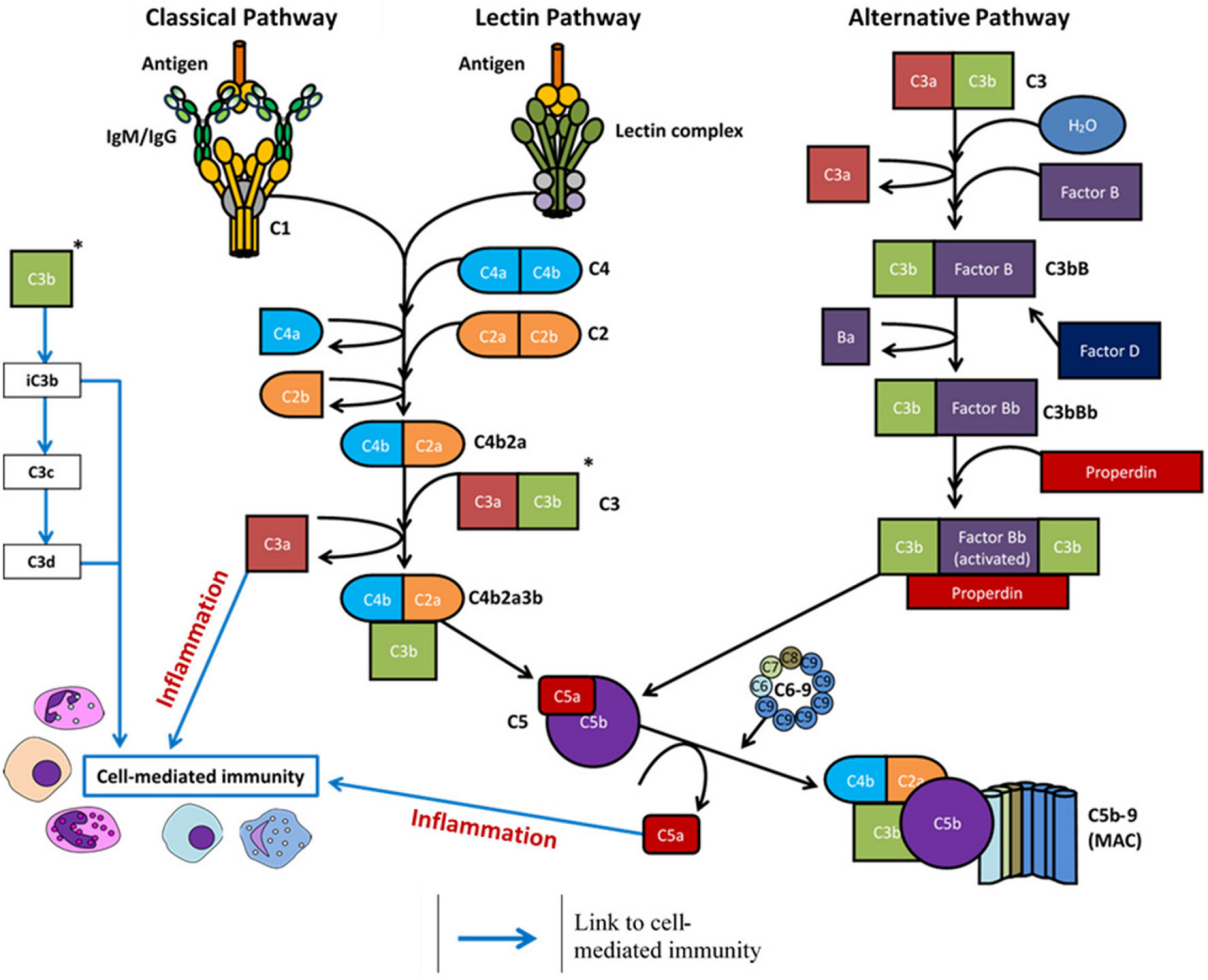
Overview of the complement system following activation via antigen (classical and lectin pathways) or spontaneous hydrolysis (alternative pathway). Complement activation eventuates in formation of the membrane attack complex (MAC) and the cleavage products regulate inflammation (C3a and C5a) and cell-mediated immunity (C3a, C3c, C3d, C5a).

The C1q molecule is an assembly of six heterotrimers, each containing three chains (C1qA, C1qB, and C1qC) with a central collagenous stem and a globular head at the C-terminus. In native plasma, the C1q molecule forms a calcium-dependent complex with two C1r and two C1s serine proteases to form the C1 complex (25). Ligand recognition and binding via the C1q molecule in the C1 complex induces a conformational change and autoactivation of the C1r_2_s_2_ tetramer to activate the classical complement pathway (11, 12). Activated C1s cleaves complement proteins C4 and C2 into active fragments C4b and C2a, along with an inactive fragment (C2b), and a protease-activated receptor (PAR)1/PAR4 ligand (C4a) which increases endothelial cell permeability (26). Non-covalent binding of C4b and C2a forms the classical pathway C3 convertase, C4bC2a, responsible for cleavage of C3 into C3a (anaphylatoxin) and C3b (active component of the convertase). The newly formed C4bC2aC3b complex is a C5 convertase formed from either the classical or lectin complement pathway activation, which cleaves the C5 molecule into C5a (anaphylatoxin) and C5b. C5 proteolysis and the successive steps are then the same for each of the three complement pathways - C5b is deposited onto the activating surface and subsequent, irreversible binding of C6, C7, C8, and multiple copies of C9 to permeate the cell surface membrane and form the MAC (11, 12, 27). All three complement pathways are summarized in Figure 1.

### Lectin Complement Pathway

The lectin complement pathway follows the same enzymatic cascade as the classical pathway but is distinct in the antigens and proteases required for its activation. The lectin pathway is activated in response to invading pathogens via direct binding of PAMPs by various C1q-like lectins, complexed with mannose-binding lectin (MBL)-associated serine proteases (MASPs)-1/2/3. These C1q-like activators are MBL, ficolin-1 (M-ficolin), ficolin-2 (L-ficolin), ficolin-3 (H-ficolin), and collectin-11 (CL-11) (28–30). In humans, MBL is typically present as a trimer, tetramer, pentamer, or hexamer and these oligomeric structures influence its carbohydrate binding properties (31, 32). Each monomeric subunit in the complex is a homotrimer with each polypeptide containing a cysteine-rich region at the N-terminus, followed by a collagen-like domain, a neck region, and a carbohydrate recognition domain capable of binding specific sugars present on pathogenic surfaces i.e., *N*-acetyl-D-glucosamine and D-mannose (33, 34).

Similar to MBL, multimeric ficolin complexes are assembled through homotrimer subunits with cysteine-rich N-terminal segments which form interchain disulphide bonds, followed by collagen-like regions, but they are unique in their ability to bind distinct carbohydrates via their C-terminal fibrinogen-like domains (35–37). Ficolin-1 is predominantly synthesized in monocytes and granulocytes where it can be found present on their surface or extracellularly in native human plasma. Ficolin-2 is synthesized in the liver and secreted into the bloodstream where it can bind to various acetylated structures and sugars via three inner binding sites (38). Ficolin-3 is synthesized in the liver and lungs and is present in native plasma at a higher concentration than ficolins-1/2, although less is known about its functional capabilities (39). Collectin-11 (CL-11) can form heterotrimeric complexes with collectin liver 1 (CL-10) in serum and can also associate with MASPs to activate the lectin complement pathway (40).

Once one or more of these lectins have complexed with MASP-2 and bound their specified target, MASP-2 then cleaves C4 and C2 to form the C3 convertase (C4bC2a complex). Following the proteolytic cleavage of C3, the lectin complement pathway follows the same catalytic process as the classical pathway (Figure 1) (2). The roles of MASP-1 and MASP-3 in this pathway are relatively ambiguous (29, 41). MASP-1 is capable of cleaving complement component C2 and, to a much lower extent, components C3 and C4 (29, 42), whilst MASP-3 may have a negative regulatory role of the lectin pathway through downregulation of MASP-2 cleavage activity (43).

### Alternative Complement Pathway

The alternative pathway does not require the specific protein-protein or protein-carbohydrate interactions seen with the other two pathways. Under normal physiological conditions, ~1% of C3 per hour undergoes spontaneous hydrolysis as the internal thioester bond is cleaved to produce C3(H_2_O). This process is augmented in the presence of various surfaces which lack complement regulatory proteins, as electrostatic and/or hydrophobic interactions adsorb C3 to the surface to induce conformational changes (44, 45). C3(H_2_O) can then bind factor B to induce another conformational change, as factor B is then cleaved into two components by factor D: Ba (which dissociates from the complex) and Bb (which remains bound in the complex). The protein complex C3bBb is the alternative pathway C3 convertase, which is stabilized through the binding of properdin to produce C3bBbP and can cleave further C3 molecules through the serine protease activity of fragment Bb. The alternative pathway therefore has the potential to both activate and enhance complement activity through an amplification loop; cleaved C3 components produce C3 convertases which cleave further C3 molecules (46, 47). Cleavage of C3 also yields C3a and C3b, where C3b remains bound in the complex to form the alternative pathway C5 convertase, C3bBbPC5b, and C3a acts as an anaphylatoxin. The rest of the complement cascade is then identical for all pathways (Figure 1) (48, 49).

Although complement activity typically occurs via the three pathways described, less conventional mechanisms of activation and immune modulation can occur, and have been discussed in a recent review (50). Typically, properdin is described as a positive regulator of the alternative pathway through stabilization of the C3 and C5 convertases. But its functions extend beyond this, including complement activation via direct antigen recognition of invading pathogens and apoptotic/necrotic cells (51–53), and as a potential ligand for NKp46-mediated natural killer cell activation and subsequent secretion of X-chemokine ligand 1 (54). Similarly, C3 and its cleavage products are often described as extracellular components, yet they can have intracellular signaling roles to eliminate pathogens, alter cytokine signaling profiles and induce Th1 responses (6, 55–57).

### Complement Protein Expression and Regulation

**Figure 2:**
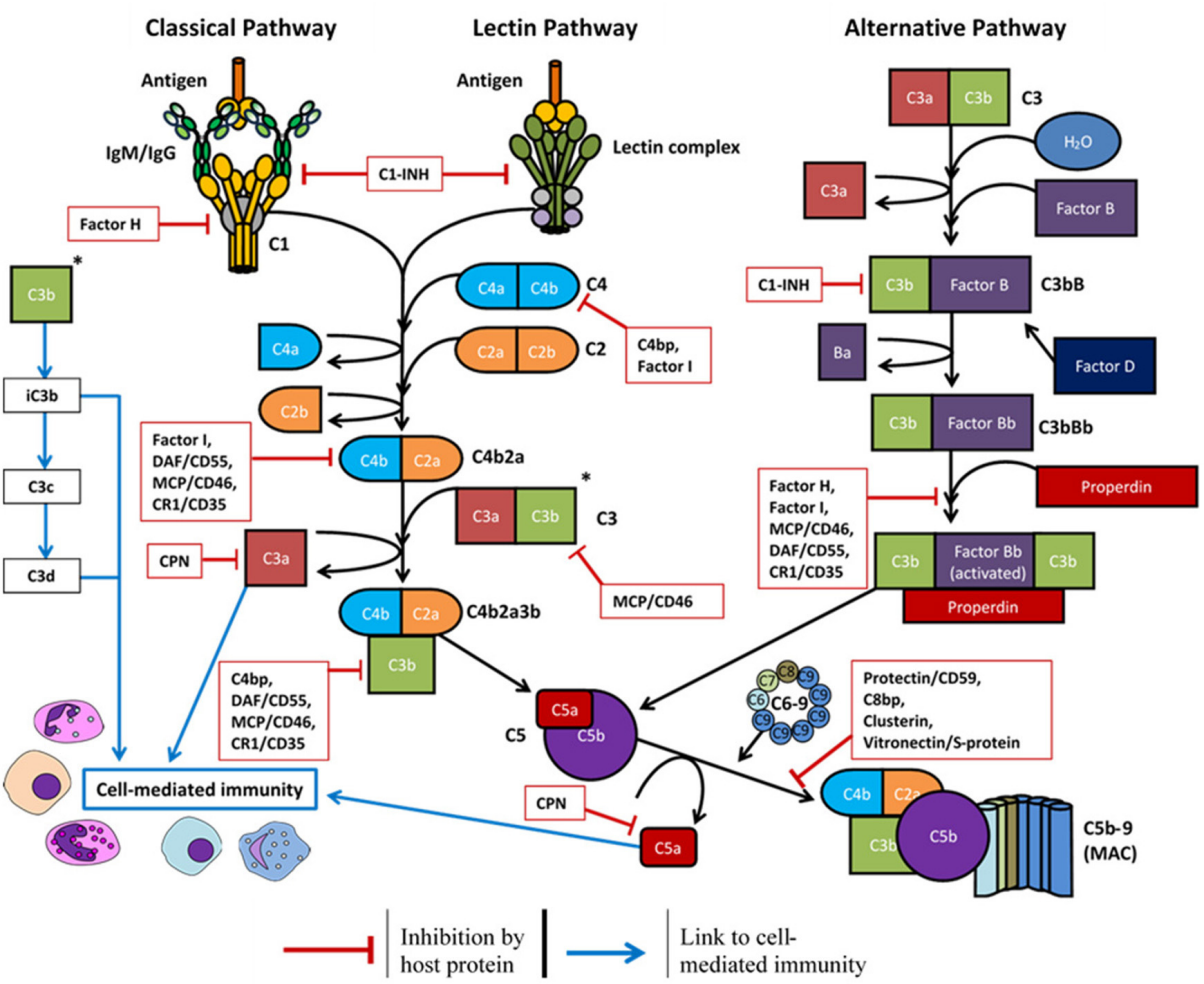
Overview of the complement system and the host soluble/membrane-bound regulatory proteins (red boxes): C1-INH, C1-inhibitor; C4bp, C4-binding protein; C8bp, C8-binding protein; CPN, carboxypeptidase-N; CR1, complement receptor 1; DAF, decay-accelerating factor; MCP, membrane cofactor protein.

Most complement proteins are primarily synthesized in the liver and secreted into the bloodstream; this process is rapidly upregulated during infection (58). Complement proteins can also be produced by epithelial cells (59), endothelial cells (60), and circulating immune cells such as dendritic cells, granulocytes, macrophages, and monocytes (61, 62). Local production of complement also occurs in immune privileged sites including the brain (63), eyes (64), and testis (65). To regulate this activity and prevent damage to healthy cells and tissues, many regulatory proteins are primarily expressed as either soluble plasma proteins or cell-surface receptors (Figure 2 and Table 1).

**Table 1:** Overview of key complement regulatory proteins and receptors, their location within plasma and on circulating immune cells, and their roles within the complement system of humans.

**Complement receptors**				
**Complement protein receptors**	**Examples of immune cell expression**	**Host complement protein target(s)**	**Role(s) within the complement system**	**References**
A_2_β_1_ integrin	Mast cells	C1q	1) Mast cell activation and cytokine secretion	(66)
C1q-Rp or C1qR1 or CD93	Dendritic cells, monocytes, neutrophils	C1q	1) Potentially modulate C1q-dependent phagocytosis	(67–69)
C3a receptor (C3aR)	Astrocytes, basophils, dendritic cells, eosinophils, macrophages, mast cells, monocytes, neutrophils, T cells	C3a	1) Enable broad biological functions of C3a	(70–78)
C5a receptor (C5aR) or CD88	Basophils, dendritic cells, eosinophils, mast cells, monocytes, neutrophils, natural killer cells	C5a	1) Enable broad biological functions of C5a	(70–72, 79–82)
cC1qR or calreticulin or collectin receptor	Ubiquitous, excluding erythrocytes	C1q collagen-like region, CD91	1) Complex with CD91 to enhance phagocytosis of C1q-coated particles	(83, 84)
CD91 (LRP-1) or α2 macroglobulin receptor	Astrocytes, dendritic cells, fibroblasts, monocytes	C1q and cC1qR	1) Complex with cC1qR to enhance phagocytosis of C1q-coated particles	(85, 86)
Complement receptor 1 (CR1) or CD35	B cells, basophils, erythrocytes, follicular dendritic cells, monocytes, neutrophils, T cells	C1q, C3b, C4b	1) Bind opsonised C3b particles to enhance phagocytosis 2) Removal of immune complexes via erythrocytes 3) Enhance B-cell activation, production of antigen-specific antibodies proliferation, and 4) Protect host epithelial cells from complement activity	(79, 87–94)
Complement receptor 2 (CR2) or CD21	B cells, follicular dendritic cells, T cells	Polymerized iC3b, C3dg, C3d	1) Enhance B-cell maturation through recognition of C3d-coated antigens and co-ligation with B-cell receptors	(95–97)
Complement receptor 3 (CR3) or CD11b/CD18 or MAC1	Basophils, dendritic cells, macrophages, monocytes, neutrophils, natural killer cells	iC3b	1) Mediate phagocytosis of C3b-bound targets 2) Suppress dendritic cell stimulation	(79, 97–99)
Complement receptor 4 (CR4) or CD11c/CD18	Basophils, dendritic cells, macrophages, monocytes, neutrophils	iC3b	1) Mediate phagocytosis of C3b-bound targets	(79, 97, 99)
Complement receptor of the immunoglobulin family (CRIg)	Kupffer cells and macrophages	C3b, iC3b	1) Phagocytosis of C3-opsonised particles in circulation	(100)
gC1qR	B cells, immature dendritic cells, macrophages, mast cells, monocytes, neutrophils	C1q globular heads	1) Mediate neutrophil and immature DC chemotaxis 2) Mediate C1q-induced immune functions	(101–104)
GPR77 or C5L2	Adipose tissue, leukocytes, natural killer cells	C5a	1) Generally considered to be a non-signaling receptor	(82, 105)
**Complement regulators**				
**Complement protein regulators**	**Examples of protein location**	**Host complement protein target(s)**	**Role(s) within the complement system**	**References**
CD46 or membrane cofactor protein (MCP)	Ubiquitous on cell surfaces excluding erythrocytes. Soluble form also circulates in tears, plasma and seminal fluid	C3b and C4b	1) Cofactor for factor-I mediated C3b and C4b inactivation 2) Inhibit C3b deposition 3) Co-stimulator for Th1 IFN- γ production	(106–109)
C1-inhibitor (C1-inh)	Soluble form in plasma	C1r, C1s, MASP-1, MASP-2, C3b	1) Inhibit C1r and C1s of the classical pathway 2) Inactivate MASP-1 and MASP-2 of the lectin pathway 3) Bind C3b to inhibit factor B binding	(41, 110)
C4-binding protein (C4bp)	Soluble form in plasma	C4b, C3b and C-reactive protein	1) Present C3b for Factor I cleavage 2) Accelerate the decay of the classical C3/C5 convertase 3) Act as a cofactor for factor I inactivation of C4b	(111–113)
C8 binding protein (C8bp)	Peripheral blood cells and muscle cells of myocardial tissue	C8	1) Prevent MAC formation	(114)
Carboxypeptidase-N/R (CPN/CPR)	Soluble form in plasma	C3a, C5a	1) Inhibit C3a and C5a through cleavage of carboxy-terminal arginine residues	(115)
CD55 or decay-accelerating factor (DAF)	Ubiquitous	C3b, C4b	1) Destabilize C3 and C5 convertases 2) Regulate T cell immunity	(116–118)
CD59 or protectin	Ubiquitous	C5b-8 and C9	1) Prevent MAC formation 2) Regulate B-cell, T-cell, NK cell responses	(119–122)
Clusterin	Soluble form in plasma	C7, C8, C9	1) Prevent lytic activity of the MAC	(123)
Factor H	Soluble form in plasma and adherence to cell surfaces expressing polyanions	C3b	1) Accelerates decay of alternative pathway C3 convertase (C3bBb) 2) Factor I cofactor for cleavage and inactivation of C3b 3) Prevents further C3b deposition on cell surface membranes 4) Competes with C1q to certain binding sites	(111, 124–126)
Factor I	Soluble form in plasma	C3b, iC3b, and C4b	1) Cleavage of C3b and C4b components	(127, 128)
Properdin	Soluble form in plasma	C3bBb	1) Stabilize alternative pathway C3 convertase (C3bBb) 2) Pattern recognition molecule from complement activation	(51, 129)
Vitronectin or S protein	Soluble form in plasma	C5b-7	1) Block membrane binding of C5b-7 2) Prevent C9 polymerization	(130)

Complement regulatory proteins may be unique to a certain pathway or influence the downstream activity of all three pathways. Factor H, factor I, and properdin are unique to the alternative pathway. Factor H is both a soluble and cell-surface membrane regulator (124) which accelerates the decay of the C3 convertase (C3bBb) to reduce complement deposition (125), and it functions as a Factor I cofactor to cleave C3b and C4b components (111). Properdin is a positive regulator of the alternative pathway which stabilizes the C3 convertase (C3bBb) and promotes its association with further C3b molecules (129). The C1-inhibitor (C1-inh) is a negative regulator of all three pathways. C1-inh competes with factor B to limit activation of the alternative pathway (110), inhibits C1r and C1s to prevent classical pathway activation (41), and inactivates MASP-1 and MASP-2 to prevent lectin pathway activation (41).

Further proteins are required to regulate the downstream complement activity. Carboxypeptidase-N/R regulates the anaphylatoxin activity of C3a and C5a via cleavage of their arginine residues (115). C8 binding protein (114), clusterin (123), and vitronectin/S protein (130) are all soluble proteins which prevent the complete assembly of the MAC. CD46/membrane cofactor protein (106, 107), CD55/decay-accelerating factor (116, 117), and CD59/protectin (119) are ubiquitously expressed on the surface of host cell surface membranes to protect the cell from complement deposition.

## Antiviral Activity of the Complement System

One of the key functions of the complement system is to assist in the killing and containment of invading pathogens, including bacteria (131), fungi (132), protozoa (133), and viruses (134). Previous reviews have discussed various evasion mechanisms adopted by viruses to dysregulate or evade this complement activity (135–138). This knowledge may then be exploited and has previously identified novel methods for vaccine and therapeutic intervention—an area which has not been extensively discussed in the previous reviews.

**Figure 3:**
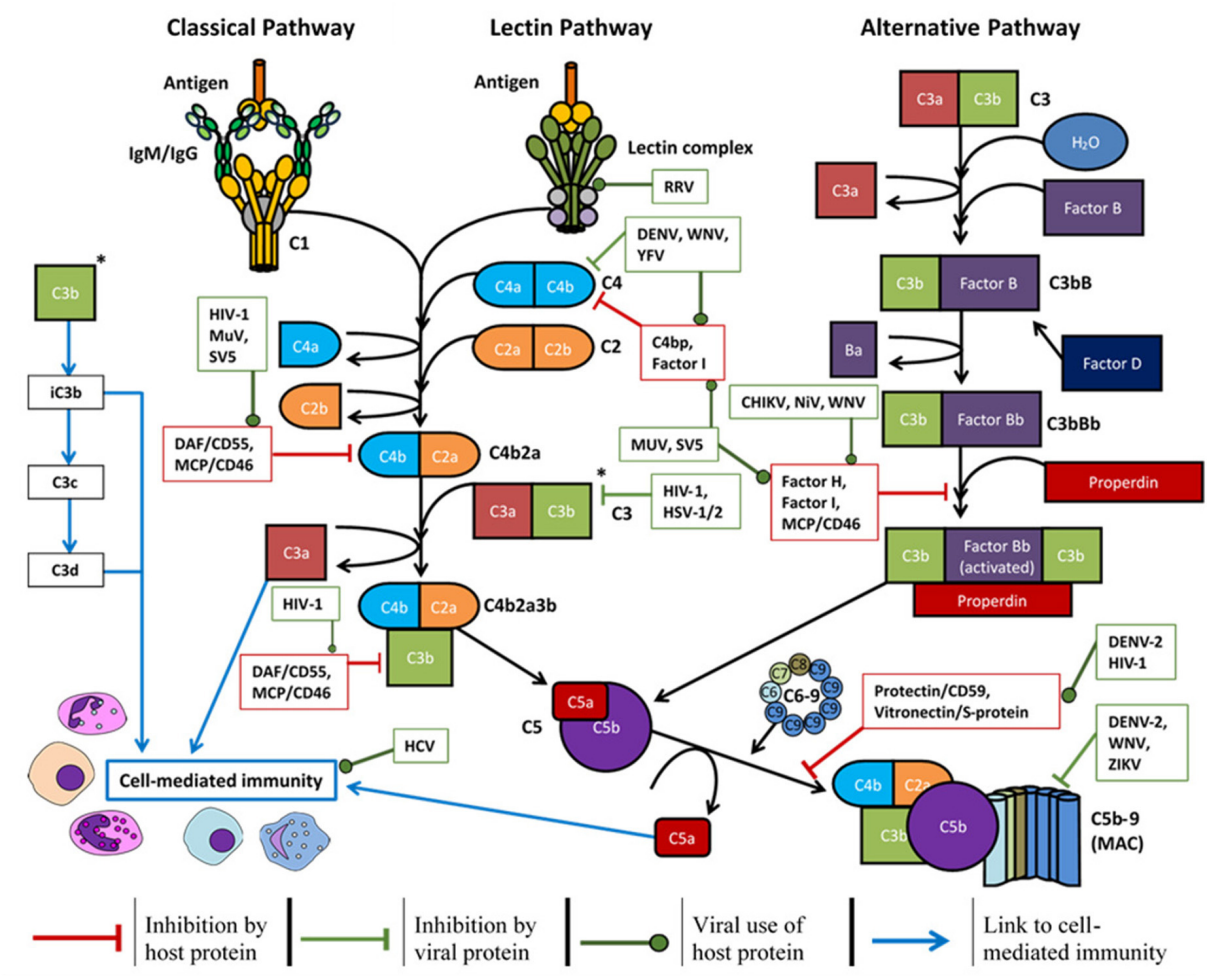
Overview of the complement system, host soluble/membrane-bound regulatory proteins influenced by the viruses mentioned (red boxes), and the regulation exerted by certain viruses (green boxes) to promote survival: CHIKV, chikungunya virus; DENV, dengue virus; HCV, hepatitis C virus; HIV-1, human immunodeficiency virus-1; HSV-1/2, herpes simplex virus-1/2; MuV, mumps virus; NiV, Nipah virus; RRV, Ross River virus; SV5, simian virus 5; WNV, West Nile virus; YFV, yellow fever virus; ZIKV, Zika virus.

The antiviral mechanisms of complement have been divided into four main sections in this discussion; physiologically however, each section is not exclusive as they work together to form a complete system. Briefly, complement deposition on a virion can block interactions with host cell receptors, aggregate virus particles, signal intracellularly to induce an antiviral state, and enhance phagocytosis (3, 6, 139). This can lead to formation of the MAC and lyse lipid membranes of enveloped viruses (140) or lyse infected host cells expressing viral antigens (141). Activation of the complement system also produces pro-inflammatory anaphylatoxins (C3a, C5a, and putatively C4a) which can enhance phagocytosis and in some cases, worsen disease symptoms (142). Lastly, these processes can enhance the adaptive immune response to viral antigens, induce a Th1 response (56), modulate Treg and Th17 responses (143), prolong B-cell memory and significantly increase antigen-specific antibody titres (144). Many of these functions may be evaded or manipulated by different viruses (shown in Figure 3) and such examples are provided throughout.

### Complement Deposition and Virus Opsonisation

All three complement pathways can lead to virus opsonisation and complement deposition following activation. The outcome of this response largely depends on the infectious agent and could enhance viral infection, suppress viral infection, or be dysregulated by the expression of some viral proteins.

The MBL protein of the lectin pathway can interact with numerous viral antigens and have varying effects on neutralization or viral enhancement. MBL can directly bind the Ebola virus (EBOV) glycoprotein (GP). High doses of MBL, relative to other complement proteins, can enhance EBOV-GP pseudotyped virus infection into primary human macrophages and human monocyte-derived macrophage cell lines (145). However, MBL opsonisation of the EBOV-GP can neutralize EBOV-GP-pseudotyped virus by preventing cell interactions via DC-SIGN (3). MBL has also been successfully used as a rescue therapy in 40% of mice when administered at supra-physiological levels, 24 h post-lethal challenge with a mouse adapted EBOV strain (146). So, in the context of EBOV infection, the effects of MBL appear to be dependent on the cellular target and the relative concentrations of other complement protein components.

MBL has also been shown to bind the HIV-1 protein, gp120. This interaction was sufficient to neutralize cell-line adapted HIV infection of CD4+ H9 lymphoblasts (134). A later study reported a similar finding, although much higher concentrations of MBL were required to achieve the same level of neutralization (50 μg/mL rather than 1 μg/mL of MBL), and these findings were not replicated when using HIV primary isolates or other cell lines for infection. In the later study, MBL was shown to be sufficient for virus opsonisation but not neutralization (147). This highlights an important consideration for *in vitro* studies when investigating complement and pseudovirus interactions, as small method variations can yield significantly different results. Where possible, *in vivo* experiments can help validate this work and address possible discrepancies. Further possible implications of MBL during HIV infection have been shown in a study of single nucleotide polymorphisms (SNPs). SNPs in the *MBL* gene which result in low serum concentrations of MBL were associated with increased risk of HIV infection and poorer prognosis following AIDS diagnosis (148).

Downstream from MBL binding, complement components are deposited on HIV virions which increase viral uptake and internalization into dendritic cells (DCs). Both complement-opsonised and complement-free HIV binding was reduced through the blockage of C-type lectins, integrins and CD4. However, the use of individual blockers showed that complement-opsonised HIV utilized β1- and β2-integrin for binding and uptake, whereas complement-free HIV utilized β2- and β7-integrin (149). A similar observation has been reported for herpes simplex virus (HSV)-2 during the infection of human DCs. Complement deposition and interactions with complement receptor 3 (CR3) enhanced HSV-2 infection of immature DCs and increased the production of new virus particles, whereas complement with the use of neutralizing antibodies significantly reduced infection (150). This highlights another important point with regards to *in vitro* investigations of complement and viral infection. Plasma is often heat-inactivated for use in cell culture to overcome concerns of complement-mediated cytotoxicity. Consequently, investigations of virus-host cell interactions may overlook important complement-mediated interactions that would normally be present during infection.

The varied effects of MBL opsonisation during viral infection have also been described for severe acute respiratory syndrome coronavirus (SARS-CoV). Multiple studies have shown the potential for MBL to bind immobilized SARS-CoV or the SARS-CoV spike protein (151, 152). This interaction was shown to be dependent on a single N-linked glycosylation site of the spike protein and this binding could prevent spike protein interactions with DC-SIGN but not the angiotensin-converting enzyme 2 (ACE2) receptor or cathepsin-L (152). Ip et al. showed that MBL binding to immobilized SARS-CoV could also inhibit SARS infection into fetal rhesus kidney cells and enhance deposition of C4 (151). However, Leth-Larsen et al. did not observe any interactions between MBL and SARS-CoV spike protein in their study (153). Similar to HIV, several studies have found a significant difference of *MBL* SNPs associated with lower or deficient MBL serum levels in SARS patients compared to healthy Chinese population control groups (151, 154), and a reduction of MBL protein concentrations in SARS patient sera (151). However, one other study observed no significant correlation of MBL-deficient SNPs in SARS patients compared to healthy Chinese population control groups (155). The role of MBL in SARS-CoV infections appears conflicted but could be significant. As later discussed, the downstream effects of complement activation do significantly influence symptoms of coronavirus infections.

Other complement proteins and downstream products of its activation can opsonise virus particles. For DENV and West Nile virus (WNV), neutralization of the virions occurs in a C3 and C4 dependent manner following MBL binding. For WNV, neutralization was achieved independent of downstream C5 and therefore did not require formation of the MAC (156). For Simian virus 5 (SV5), complement-mediated neutralization is predominantly achieved through C3 deposition and the formation of virion aggregates, rather than virion lysis. For the closely related Mumps virus (MuV) however, the opposite effect is observed with few aggregates formed but greater susceptibility to complement lysis (157). Similarly, complement activation in the presence of influenza A virus causes virion aggregation and opsonisation of the hemagglutinin receptor. Although to achieve neutralization, IgM antibodies and activation of the classical pathway is required (139).

For Chandipura virus (CHPV), the alternative pathway and factors C3, C5, and factor B were required for complement-mediated virus neutralization in absence of C8 or antibodies (158). A different study utilized antibodies to observe classical pathway activation and reported that C1q, C3, and C4 were essential components for neutralization, but this was independent of factor B and C8 (159). The discrepancy of the importance of Factor B for CHPV neutralization could depend on the presence of antibodies and the classical pathway.

As mentioned previously, complement opsonisation of virions can enhance infection through interactions with complement receptors on host phagocytic cells (149, 160). However, some complement proteins can have a protective intracellular function as well, which is independent of cell-type (6). Enveloped viruses may naturally evade the intracellular functions of complement, as the protein deposition would occur on the lipid membrane. So, for viral entry via membrane fusion or endocytosis, it is expected that the complement-opsonised viral envelope would be left on the host cell surface membrane or endosome plasma membrane. This has been demonstrated *in vitro* using respiratory syncytial virus (RSV), an enveloped virus which enters the cell via membrane fusion, where complement intracellular signaling was absent following infection (6).

The intracellular immune function of complement has a better-defined role for non-enveloped viruses, although the area of intracellular complement immunity is still relatively new (161). In a C1-dependent manner and independent of downstream complement activity, C4 deposition on the capsid of non-enveloped human adenovirus 5 has been shown to contain the virus within the endosome, by blocking the fiber shedding and protein VI exposure mechanisms required for capsid disassembly (162). The use of an adenovirus type 5 vector (AdV) also showed that intracellular sensing of complement could inhibit infection and degrade the virus particle (6). A comparison of complement-coated AdV to AdV only, showed that intracellular C3 signaling induced the activation of pro-inflammatory cytokines (IFN-β, IL-6, IL-1β) through NF-κB, interferon-regulatory factor (IRF), and activating protein-1 (AP-1) transcription factor activation. Intracellular C3 sensing was shown to be mitochondrial antiviral-signaling protein (MAVS)-dependent, and independent of PAMPs and pattern recognition receptors. Sensing of complement-coated AdV also targeted the virion for degradation by valosin-containing protein (VCP) and the proteasome. C3-mediated signaling could induce an antiviral state in previously uninfected cells, as the supernatant from complement-coated AdV infected cells was able to protect uninfected HeLa cells from infection with interferon-sensitive Sindbis virus. Lastly, some viruses have evolved evasion mechanisms to overcome the complement-mediated intracellular immune response. Rhinoviruses and polioviruses were shown to inhibit the intracellular C3 complement signaling mechanism through the expression of a cytosolic 3C protease to degrade C3 (6).

Discussed in more detail below, some viruses encode proteases which enhance degradation of the C3 convertase to prevent further complement deposition or MAC formation. This can protect the virion from complement opsonisation and viral lysis.

### Viral/Infected Cell Lysis and Evasion

Following complement deposition and opsonisation, the complement cascade can progress to assembly of the MAC. MAC formation can perturb and lyse lipid membranes of enveloped viruses or destroy infected cells expressing viral antigens to reduce viral load (4, 5, 140, 141). Again, viral proteins can be expressed to dysregulate and evade this response.

Zika virus (ZIKV) can lead to classical pathway activation via formation of antigen-antibody complexes or through direct binding of C1q. For ZIKV derived from insect cell lines, this interaction resulted in MAC formation and a reduction of viral titres *in vitro*. However, ZIKV derived from human cell lines were more resistant to complement mediated neutralization (4). ZIKV and other Flaviviruses (including yellow fever virus (YFV), DENV, and WNV) express and secrete the non-structural protein 1 (NS-1) to regulate complement activity. The NS1 protein has a wide variety of functions in complement regulation which include: antagonism of C4 (163), recruitment of host C4 binding protein (164), recruitment of host factor H (165), recruitment of host vitronectin and inhibition of C9 polymerisation (166). However, the DENV NS1 protein is also capable of complement activation and the resulting soluble C5b-9 complexes have been found to correlate with disease severity in patients with dengue shock syndrome (167). This discrepancy was addressed with the possibility that relative IgM, C4 and soluble NS1 concentrations in plasma, at different sites of infection, could influence the extent of inhibition and therefore have varied effects on complement activity (163).

Similarly, Nipah virus (NiV) exhibits factor I-like activity, either through acquisition of factor I host protein or through inherent protease activity. Unlike soluble factor-I, NiV exhibits no capacity for C4b cleavage and showed no significant cleavage of C3b with a CD46 cofactor, despite its integration in the NiV lipid membrane. However, NiV is capable of C3b cleavage into iC3b with factor I cofactors (factor H and soluble CR1) to protect against virus neutralization (168). Chikungunya virus (CHIKV) also exhibits factor I-like activity, likely of viral origin and dependent on host factor H concentrations, to cleave C3b into inactive iC3b and resist complement-mediated neutralization (169). MuV, SV5, and HIV-1 can all recruit host cell CD46 into the viral lipid membranes during the budding process to protect from complement deposition and neutralization (170, 171). HIV-1 also incorporates glycosyl phosphatidylinositol-anchored CD55 and CD59 for further protection from complement mediated neutralization (170). Conversely, complement deposition has been shown to enhance HIV-1 infectivity into peripheral blood mononuclear cells through interactions with complement receptors (160, 172). This highlights the complexity of complement and viral interactions with dualistic mechanisms, which has previously been reviewed in the context of HIV-1 infection (173).

Infected host cells which present viral antigens on the cell surface membrane can activate the classical pathway, as the antigens bind IgM/IgG to induce complement dependent cytotoxicity (CDC). The infected cell is then lysed via the MAC in an attempt to reduce viral load. For Influenza A virus infection, complement-dependent lysis (CDL) monoclonal antibodies can cross-react with H1 and H2 hemagglutinin subtypes for broader protection than neutralizing monoclonal antibodies (141). Similarly, broadly neutralizing anti-HIV-1 antibodies can bind the viral envelope protein expressed on infected primary lymphocytes to initiate complement deposition. The deposition does not result in a rapid lytic effect but neutralizes viral spread to further cells (174). For HSV-1 and HSV-2, the glycoprotein C (gC)-1 is expressed to protect virions and infected cells from complement mediated neutralization. The gC-1 protein binds C3, C3b, and C3c to prevent subsequent binding of C5 or properdin. Modification of gC-1 on HSV infected cells can therefore increase their susceptibility to antibody neutralization and CDC (5, 175).

### Promotion of Inflammation/Chemotaxis

Some of the cleavage products from complement activation can function as anaphylatoxins and have broader immune regulatory functions. Primarily, cleavage products C3a and C5a can be generated via all three pathways and act as potent immune regulators, whilst C4a is generated via the classical and lectin pathways only (7). The role of C4a as an anaphylatoxin is disputed as it currently has no known anaphylatoxin receptor associated with its activity (176). However, it does function as an effector protein that is derived from complement activation, which enhances endothelial cell permeability and increases stress fiber formation via PAR1 and PAR2 (26). The roles of C3a and C5a are better described as anaphylatoxins, with the latter demonstrating higher stability and broader biological activity. C5a recruits neutrophils to the site of inflammation and both C3a and C5a can recruit: eosinophils, fibroblasts, macrophages, mast cells, and monocytes (70–72, 177–179). These two anaphylatoxins demonstrate a large functional overlap but each have their own discrete functions. To varying degrees, both are capable of stimulating the production of pro-inflammatory mediators from monocytes and macrophages via inflammasome-caspase-1 activation (180, 181). Both can induce the degranulation of mast cells (182–185), basophils (186–188), and eosinophils (189). Both induce respiratory bursts in eosinophils (190) and neutrophils, although only C5a shows chemotactic activity for neutrophils whereas C3a may actually prevent neutrophil mobilization from the bone marrow (191). Further, only C5a can stimulate respiratory bursts in macrophages (192).

The activity of C3a and C5a is mediated via binding to two main G-protein coupled receptors; C3aR or C5aR, respectively (193). A secondary, non-G-protein coupled receptor (C5L2) has been shown to bind C5a and potentially regulate its biological functions *in vitro*, although its primary functions are not yet clear (105). These receptors are widespread across different cell types including both myeloid cells and non-myeloid cells (e.g., astrocytes, microglia, hepatocytes, endothelial and epithelial cells) to produce various biological functions dependent on the cell type (194, 195). C3a and C5a activity is further regulated by the enzyme carboxypeptidase-N. Carboxypeptidase-N cleaves the carboxy terminal arginine amino acid of these anaphylatoxins to generate products with greatly reduced (C5a-desArg) or absent (C3a-desArg) anaphylatoxin activities (196). The cleavage product C5a-desArg retains some chemotactic activity to recruit distant immune cells (188, 197) whilst C3a-desArg can function as a hormone for lipid metabolism (198). Beyond their roles in chemotaxis, C3a and C5a have been associated with: the induction of smooth muscle contraction (199), regulation of vasodilation (200), an increase in vascular permeability (201), and the production of various cytokines including IL-1β, IL-8/CXCL-8, CCL5, IL-6, TNFα (180, 193).

During viral infection, excessive complement activation leading to a strong pro-inflammatory response is often associated with more severe disease symptoms. The negative impact of complement activation has been associated with more severe symptoms during SARS-CoV and MERS-CoV infections. Infection of C3 deficient mice with SARS-CoV revealed that the loss of complement activity resulted in milder disease outcomes (202). Compared to the wild-type, the C3 deficient mice showed: no significant weight loss, improved respiratory function, reduced lung pathology, and lower levels of inflammatory cytokines and chemokines (202). Proteomic analysis has shown that a product of complement activation, C3c α chain, was significantly higher in SARS-patient sera compared to non-SARS patient sera (203). Similarly, increased concentrations of C5a and C5b-9 were observed in sera lung tissues of *hDPP4*-transgenic mice challenged with MERS-CoV. The subsequent use of a C5aR antibody to prevent C5a functional activity resulted in reduced tissue damage and a lower viral load (204). Cytotoxic effects of complement may also occur post-SARS-CoV infection. Autoantibodies elicited 1-month after infection against epithelial and endothelial cells can mediate complement-dependent cytotoxicity and enhance lysis against A549 cells and human placenta endothelial cells (205).

In patients with severe DENV infection and dengue shock syndrome, overactivity of the alternative pathway has been reported with increased levels of NS1, C5a, and sC5b-9 in pleural fluids, which likely contribute to the symptoms of increased vascular permeability (167, 206). In DENV infected cells, indicators of the alternative complement pathway are upregulated, with a relatively higher concentration of Factor B to factor H proteins and increased cell surface C3b deposition (206).

In mice infected with Ross River virus (RRV), complement activation products have been identified in serum and inflamed tissues. Similar observations have been made in the synovial fluid of RVV-infected patients. In C3 knockout mice, the signs of severe disease and tissue damage from RVV infection were diminished compared to wild-type, which suggests complement promotes RRV-induced inflammation (207). RRV infected cells express the viral E2 protein which is glycosylated with N-linked glycans. E2 N-linked glycans are antigens for MBL and can activate complement via the lectin pathway, which results in greater inflammation and tissue damage during RVV infection (208, 209).

### Complement Enhancement of Adaptive Immunity

Complement activation also plays an important role in linking the innate and adaptive immune responses. This interaction can enhance the production of antigen-specific antibody titres and shape the T-cell response to target viral pathogens more efficiently. The importance of complement in the regulation of T-cell immunity has previously been reviewed (50, 210).

Cognate and co-stimulatory interactions (CD80-, and CD86-CD28, and CD40-CD40 ligand) between antigen presenting cells (APCs) and T-cells results in the local production of C3, factor B, factor D, and C5. Receptors C3aR and C5aR are also upregulated on the T-cell surface whilst production of decay accelerating factor (DAF) is down-regulated. The local production of complement components from immune cells enables signaling via C3aR and C5aR in an autocrine and paracrine manner. Complement C3 can also be processed intracellularly, or internalized as C3 (H_2_O) from the alternative pathway, to increase pro-inflammatory cytokine expression from T-cells and recycled back to the T-cell surface (55, 57). A major component of C3 cleavage on the T-cell surface is iC3b. T-cell membrane bound iC3b binds to CR3 (and possibly CR4) on monocyte derived DCs to enhance T-cell proliferation (9). Absence of C3aR and C5aR leads to: reduced complement protein and receptor regulation, lack of co-stimulatory molecule expression, impaired cytokine production (IL-1, IL-23, and IL-12), an induction of an iTreg cell response, and suppression of T-cell proliferation (211–213).

Activation of both C3aR and C5aR on DCs by their respective anaphylatoxins (C3a and C5a) can mediate the production of IL-6, IL-23, the IL-12 receptor, and TGF-β1 to promote T-cell differentiation into antiviral Th1 and Th17 subsets (143). Induction of the Th1 response also depends on C3aR and CD46 activation on T-cells via their T-cell derived ligands (56). In mice infected with Influenza A virus, inhibition of the C5aR lead to a reduction in influenza-specific cytotoxic CD8^+^ T-cells (214) and C3 deficiency lead to increased viral titres and delayed viral clearance (215). C3 is also required for the production of antigen-specific CD8^+^ T-cell responses during lymphocytic choriomeningitis virus infection in mice (216). During HCV infection, the HCV core protein can interact with gC1qR on host immune cells and suppress the T-cell response. This interaction inhibits T-cell proliferation in a dose-dependent manner to downregulate CD69 activation and reduce the production of IFN-γ and IL-2 from T-cells (217–219). HCV core protein interaction with gC1qR on monocyte-derived DCs inhibits IL-12 production and promotes Th2 cytokine production to limit differentiation into Th1 cells (218). HCV core protein interaction with gC1qR on B-cells has a differential response to the one observed on T-cells and DCs, as it increases cell surface costimulatory and chemokine receptor expression and enhances B-cell proliferation (219). Furthermore, the HCV core protein exhibits intracellular functions, as it can suppress the T-cell factor-4 transcription factor required for C9 promoter activity regulation. This reduces C9 mRNA and protein levels which are required for complete MAC assembly (220).

As discussed previously, complement activation can result in C3 deposition on the surface of virions. C3 and its cleavage products can interact with the B-cell receptor and B-cell co-receptor complex (CR2/CD21 ligated with CD19 and CD81) to lower the B-cell activation threshold by several orders of magnitude. This can dramatically increase antibody titres, modulate the proliferation of mature B cells, and protect the B-cells from CD95-mediated elimination (8, 144). Immune complexes coated in C3 and C3 cleavage products covalently interact with complement receptors on follicular DCs (FDCs). The C3-coated immune complexes on FDCs are then presented to B-cells in the germinal center for optimal B-cell responses, including: antibody production, somatic hypermutation, class switching, and affinity maturation (87, 221). FDCs can then retain the C3-coated complexes within the lymphoid for extended periods of time to generate memory B-cells and promote survival (222).

Alternatively, some aspects of the complement system can suppress certain responses of adaptive immunity: stimulation of CR3 on DCs can suppress the release of inflammatory cytokines (98) and C1q-differentiated DCs demonstrate an increased phagocytic capacity but reduced expression of CD80, CD83, and CD86 required for T cell activation (223).

## Significance for Vaccines and Therapeutics

It is apparent that the complement system has important implications for virus neutralization and development of the adaptive immune response. As our knowledge of virus and complement interactions improves, this can inform novel approaches for intervention and the development of therapeutics and vaccines. One such example is the use of rupintrivir against rhinoviral infections. Rhinoviruses encode a cytosolic 3C protease which cleaves intracellular C3 to avoid the intracellular mechanisms of complement, mentioned previously. Rupintrivir inhibits the viral cytosolic 3C protease to increase susceptibility of the virus to intracellular complement immunity (6). Similarly, the use of Fab fragments could prevent the C4 inhibition of human adenovirus 5 vector for its use in adenoviral gene therapy to promote efficient transgene delivery (162).

Due to the multifaceted and complex immune functions of the complement system, direct manipulation of complement would need to be carefully considered. Inhibition of the complement system could increase susceptibility to other diseases, whilst over-stimulation could result in autoimmunity and damage to host cells. A method of complement stimulation through inhibition of the CD59 regulator has been proposed for the treatment of latent HIV-1 infection in cells. The use of provirus stimulants and a CD59 inhibitor showed a dose-response effect of cell sensitization to antibody-dependent cell-mediated lysis and reduced viral load. Aside from the target cells, no significant non-specific cytolytic effects were observed *in vitro*. CD59 protects host cells from complement activity, is ubiquitously expressed, and so its inhibition has the potential to damage host cells (224, 225). Deletion of CD59 in mice did not have a lethal outcome, however absence of the complement regulatory protein did lead to intravascular haemolysis and thrombosis (226). Treatment in the context of HIV-1 infection would be short-term however (224) and could be an exception for an otherwise incurable disease. Similar approaches have been considered for other life-threatening diseases such as cancerous conditions (227, 228).

Methods of complement inhibition have also demonstrated therapeutic benefit. Mentioned previously, excessive complement activation is associated with more severe outcomes of MERS-CoV and SARS-CoV infections. Use of a C5aR antibody to block the pro-inflammatory effects of C5a in MERS-CoV infected *hDPP4*-transgenic mice resulted in: lower concentrations of pro-inflammatory cytokines, reduced viral replication in lung tissues, reduced lung and spleen tissue damage, and a reduction of viral antigen and microglia activation in the brain (204). Excessive complement activation and similar lung pathology during SARS-CoV infection has also been observed in H5N1 influenza cases, where the use of C3aR and C5aR antagonists reduced signs of acute lung injury and viral load in H5N1-infected mice (229).

A novel coronavirus, SARS-CoV-2, has recently emerged and is the causative agent of COVID-19—an acute self-limiting disease which has the potential to progress to severe disease and death. Symptoms of severe disease involve major alveolar damage, wide-spread lung inflammation, and progressive respiratory failure (230, 231). The pathological features of lung, liver, and heart tissue in a severe case of COVID-19 greatly resembled those seen in SARS-CoV and MERS-CoV infections which are complement-mediated (202–205, 231). MBL has been shown to activate complement via binding to SARS-CoV spike protein in some studies and this could translate to the SARS-CoV-2 spike protein, which contains N-linked glycosylation sites that are targets for MBL (151, 232). Thus, the widespread lung inflammation observed in severe cases of COVID-19 could be exacerbated by excessive complement activation. Furthermore, viral infections with similar lung pathology to COVID-19 have demonstrated therapeutic benefit with the administration of complement inhibitors targeting C3a/C3aR or C5a/C5aR. This has been shown for H5N1 (229), H7N9 (233), and MERS-CoV (204) infections. So, it seems plausible that the lung inflammation in severe cases of COVID-19 is exacerbated by excessive complement activation and this pathologic inflammation could be attenuated through use of complement inhibitors.

Clinical trials are currently being conducted with the use of a C5a inhibitor, the monoclonal antibody IFX-1, which has proven to be well tolerated in 300 clinical trial participants and aims to reduce inflammation whilst preserving MAC formation (234). As SARS-CoV-2 is an enveloped virus (235), it is possible that MAC formation could have some beneficial antiviral effects. Therefore, a C5a inhibitor such as IFX-1 (InflaRX) may be favorable mechanistically over a C5 inhibitor such as eculizumab (Soliris), which is also considered for use in clinical trials (NCT04288713) (236). The IFX-1 monoclonal antibody targets a specific conformational epitope of the C5a molecule to block its anaphylatoxin activity, whilst C5b and downstream complement activity are preserved (237). Eculizumab is a monoclonal antibody which targets the C5 molecule to prevent cleavage into C5a and C5b, and therefore inhibits all downstream complement activity (238). The distinction between the two antibodies is the preservation of MAC activity which could be relevant against the enveloped SARS-CoV-2, although the effects of complement lysis and whether it occurs on SARS-CoV-2 is not yet known.

Because the efficacy and safety of eculizumab is already well characterized (239), it is logical that this would take precedence over lesser-known options for urgent clinical trials. The use of eculizumab has already proven beneficial for treatment of severe cases of COVID-19, which shows that complement is partly responsible for the symptoms in severe cases (240). It would be interesting to compare the effects of preserving the MAC during infection with the enveloped SARS-CoV-2, as it may offer an antiviral, as well as an anti-inflammatory, effect. But it is also possible that Coronaviruses have an intrinsic evasion mechanism, perhaps similar to the ones described in this review, to avoid the lytic activity of the MAC. Another important consideration could be the stage of infection for implementing complement inhibitors: maintaining complement activity may have a beneficial impact early on in infection for virus neutralization and the development of adaptive immunity, and intervention may only be required to treat excessive inflammation in severe cases.

The complement system has several important considerations for vaccine development, one example being its involvement in antibody dependent enhancement (ADE). ADE is commonly observed when non-neutralizing antibodies are present following initial priming of the immune system. Non-neutralizing antibodies can still bind the viral target with the potential to cross-link with Fc receptors, or activate complement and interact with complement receptors, to enhance viral infection of host cells (241). ADE is more commonly observed to be Fc receptor-mediated, however complement-mediated ADE has been reported for HIV-1 (242), MERS-CoV (243), and EBOV (244).

But complement activation can have a positive effect against viral infections in the presence of some non-neutralizing antibodies. Use of the non-neutralizing influenza virus M2 extracellular vaccine in mice required functional C3 to confer protection and induce effective humoral and cell-mediated immune responses (245). A similar effect has been reported for monoclonal antibodies against human cytomegalovirus (HCMV). Following the use of gB/MF59 HCMV vaccination in humans, the immune sera had enhanced neutralization potency toward HCMV in the presence of complement. Certain HCMV monoclonal antibodies rely on complement for viral neutralization, which appears distinct from CDC or virolysis, and is likely the result of blocking virus-host interactions (246). Complement activity has also been implicated for optimal protection with non-neutralizing antibody mAB-13G8 against Crimean-Congo haemorrhagic fever virus infection in adult mice (247).

In Flavivirus infections, the mechanism of ADE is predominantly shown to be Fc mediated (248). Complement has been shown to augment antibody-mediated neutralization of WNV *in vitro* (249) and the addition of C1q has been shown to lower the antibody concentrations required for WNV neutralization *in vitro*, which correlated with protective effects observed *in vivo* (250). C1q was also shown to mediate effects of ADE from Flavivirus infections in a subclass specific manner, whilst MBL, factor B, or C5 depletion had no significant effect (251). Although IgG subclasses are known to bind C1q with varying avidities, the mechanism to explain this effect on ADE has not been identified. This could highlight the importance of selecting the right antibody subclass when considering monoclonal antibody therapies.

In general, vaccines which effectively engage the complement system may gave rise to a more potent, virolytic serological response. For HIV vaccination in macaques, the presence of complement augmented virus neutralization and complement-mediated neutralizing antibody titres correlated with vaccine-mediated protection (252). Other approaches have modified vaccines to utilize aspects of the complement system for increased antigen immunogenicity, such as complement component C3d. C3d is an end-stage cleavage product from C3 activation which interacts with CR2 on B-cells, T-cells, and FDCs. When bound to an antigen, C3d can dramatically reduce the B-cell activation threshold for a stronger, more antigen-specific antibody response (8, 144, 253). CR2 on FDCs interacts with iC3b, C3d, and C3dg to enhance antibody titres and promote long-term B-cell memory development (254). C3d also bears T-cell epitopes so even with a lack of CR2 expression, the peptide can be internalized and presented on HLA II molecules to autoreactive T-helper cells and enhance antibody responses (255, 256). C3d does not interact with other components of the complement system and so the associated risks are reduced, however a large enough reduction in the B-cell activation threshold could potentially lead to antibody-mediated autoimmune responses.

C3d has been used as a vaccine adjuvant against several different viruses. DNA vaccines encoding the envelope glycoprotein of porcine reproductive and respiratory syndrome virus were more effective at increasing antigen specific neutralizing antibody titres, IFN-γ levels, and IL-4 levels when engineered with gene copies encoding the CR2 binding site of C3d in the same plasmid construct (257). Similarly, use of hepatitis E virus peptide (HEV-p179) for DNA vaccination in mice had enhanced anti-HEV-p179 antibody titres and avidity when fused with three tandem C3d copies as genetic adjuvants (258). C3d has also been used as genetic adjuvant for DNA vaccines against Newcastle disease virus and HIV-1 for increased efficacy and higher, longer-lasting antibody titres (259, 260). Fusion of C3d to target antigens is another approach for the development of safer, more immunogenic DNA vaccines. Coupling of C3d to the secretory form of Influenza virus haemagglutinin in mice provided an effective and safer mechanism for mucosal vaccination compared to the use of other adjuvants i.e., cholera toxin B subunits and *Escherichia coli* labile toxin (261). So, the use of C3d as an adjuvant can help to overcome the low immunogenicity associated with DNA vaccines, whilst maintaining their safety.

Many of the viruses discussed can activate complement, resulting in beneficial and/or detrimental effects on its survival. In the examples where viral-mediated complement activation has been more extensively studied, a viral mechanism is often identified which protects the virus from certain antiviral functions, such as the acquisition of CD46, CD55, CD59 to protect from MAC formation or the expression of a regulatory protein to inhibit the complement cascade at various points.

For the viruses which have been shown to activate complement but do not have a clear evasion/regulatory mechanism, such as MERS-CoV (204), SARS-CoV (202, 205), EBOV (3), and possibly SARS-CoV-2, it is plausible that the mechanism simply has not yet been identified. The viruses which activate complement would consequently trigger the downstream antiviral effects, both intracellularly and extracellularly. Therefore, it seems plausible that these viruses would utilize a mechanism, similar to the ones described in this review, to evade this antiviral activity and promote their survival. If such a regulatory protein or process is identified, then these may present as possible antiviral targets, similar to the targeting of the rhinovirus 3C protease with rupintrivir (6).

## Conclusion

The complex interplay between viruses and the complement system can have profound implications for protection via innate immunity and the development of effective adaptive immunity. The effects of the complement system can vary between viral infections, and even during the different stages of the same viral infection, so a clear understanding of these mechanisms is important to improve efficiency of vaccine/therapeutic development whilst mitigating risk. Such developments can also be applied for non-viral pathogens (including bacteria, fungi, protozoa) and to broader, more systemic functions of the complement system including: interferon signaling (262, 263), metabolism (264), brain development (265), and the coagulation system (266).

Components of the complement system form an ancient aspect of innate immunity in vertebrates (267) and even some invertebrates (268, 269). Therefore, many animals which act as viral hosts or reservoirs for zoonoses also have an active complement system for targeting pathogens i.e., bats (270), cows (271), deer (272), pigs (273), rabbits (274), and rats (274), which the virus may have to overcome to avoid possible antiviral activity. Further viral mechanisms of complement regulation may therefore exist which have not yet been identified and the plasticity of viral genomes could result in the emergence of novel protein regulatory functions. Identifying these novel interactions could be important for the development and augmentation of vaccines and therapeutics or even the possibility of utilizing viral-derived regulatory proteins as therapeutic complement inhibitors in other diseases (137).

The benefits from understanding complement mechanisms in viral diseases may have relevance for the current SARS-CoV-2 outbreak. Previous research has demonstrated the impact of the complement system in coronavirus infections and other diseases, and this knowledge has led to the consideration of several complement inhibitors as therapeutics for severe cases of COVID-19.

## Author Contributions

JM wrote the manuscript and designed the tables and figures. TT, SL, and MC provided guidance and revised the manuscript. All authors contributed to the article and approved the submitted version.

## Conflict of Interest

The authors declare that the research was conducted in the absence of any commercial or financial relationships that could be construed as a potential conflict of interest.
